# Antimicrobial residual effects of irrigation regimens with maleic acid in infected root canals

**DOI:** 10.1186/s40709-015-0025-4

**Published:** 2015-02-15

**Authors:** Carmen María Ferrer-Luque, Silvia González-Castillo, Matilde Ruiz-Linares, María Teresa Arias-Moliz, Alberto Rodríguez-Archilla, Pilar Baca

**Affiliations:** Department of Dental Pathology and Therapeutics, School of Dentistry, University of Granada, Campus de Cartuja, Colegio Máximo s/n, Granada, Spain; School of Dentistry, University of Granada (Spain), Granada, Spain; Department of Paediatric Dentistry, School of Dentistry, University of Granada, Campus de Cartuja, Colegio Máximo s/n, Granada, Spain; Department of Microbiology, School of Dentistry, University of Granada (Spain), Granada, Spain; Department of Oral Medicine, School of Dentistry, University of Granada, Campus de Cartuja, Colegio Máximo s/n, Granada, Spain.), Granada, Spain; Department of Preventive Dentistry, School of Dentistry, University of Granada, Campus de Cartuja, Colegio Máximo s/n, Granada, Spain

**Keywords:** Cetrimide, Chlorhexidine, *Enterococcus faecalis*, Maleic acid, Residual effects

## Abstract

**Background:**

The success of endodontic treatment depends largely on the control of microorganisms present in infected root canals. The aim of this study was to determine the residual antimicrobial activity of several final irrigation protocols with 7% maleic acid (MA) alone and combined with chlorhexidine (CHX), cetrimide (CTR) or both, in root canals infected with *Enterococcus faecalis*. Biofilms of *E. faecalis* were grown in uniradicular roots for 4 weeks. A total of 72 specimens were divided into 5 experimental groups according to the final irrigation regime used: Group 1: 2.5% NaOCl; Group 2: 7% MA; Group 3: 7% MA + 0.2% CTR; Group 4: 7% MA + 2% CHX; Group 5: 7% MA + 0.2% CTR + 2% CHX; and Control group: 0.9% saline solution. Twelve roots (2/group) that were instrumented and not infected served as negative or sterility controls. The proportion of ungrown samples over 60 days was evaluated using non-parametric Kaplan-Meier survival analysis. Differences among groups were tested using the log-rank test (*p*< 0.05).

**Results:**

The association of MA with CHX and CHX + CTR as final irrigating solutions showed the best results, 5 and 4 samples out of 12, respectively, and without differences between them (*p =* 0.928), followed by 7% MA + 0.2% CTR with 7 out of 12. The 7% MA (11/12) group showed significant differences with respect to the groups in which MA was combined with CHX (*p < 0.005*) and CHX + CTR (*p < 0.005*).

**Conclusion:**

Final irrigating solutions of 7% MA combined with 2% CHX or 2% CHX + 0.2% CTR were found to effectively improve antimicrobial root canal disinfection.

## Background

Root canal preparation by instrumentation and irrigation is an essential stage in endodontic treatment to eliminate or reduce the number of microorganisms within the root canal and to prevent bacterial recontamination in teeth with persistent apical periodontitis [1]. Intracanal cleaning and disinfecting procedures depend largely upon the chemo-mechanical effects of the irrigants. A number of chemical solutions and their combinations may be used, as no single solution is capable of dissolving organic tissue, eliminating the smear layer created during instrumentation, and preventing bacterial recolonization over a long period of time.

In an irrigation regimen, sodium hypochlorite (NaOCl) is the main solution used during and after instrumentation, given its potent antimicrobial action and ability to dissolve organic matter and necrotic tissue [2-4]. However, because it lacks residual antimicrobial activity, the regrowth of persistent microorganisms is not avoided [5,6]. In contrast, antiseptic and/or surfactant agents such as chlorhexidine (CHX) or cetrimide (CTR), with proven substantivity [7,8], have demonstrated antimicrobial residual activity when used as final irrigating solutions in different regimens [6,9,10].

Smear layer removal in root canal preparation calls for the use of chelating agents during [11] or after instrumentation [2]. The use of ethylenediaminetetraacetic acid (EDTA) followed by NaOCl has proven to reduce or eliminate *Enterococcus faecalis* biofilm in final irrigating regimens [6,12]. Maleic acid (MA), a mild organic acid, has recently been proposed as an alternative irrigating solution to EDTA, because of its ability to remove smear layer [13], its lower toxic effects [14] and greater effectiveness in eradicating *E. faecalis* biofilms as compared to EDTA or citric acid [15].

When MA is combined with CTR, it maintains the extraction —although somewhat diminished— of calcium ions from root dentin [16], thereby helping preserve the structural composition of root canal dentin [17]. This association also enhances its residual antimicrobial activity [10] and there is no precipitate formation when MA is mixed with CHX solutions [18].

A previous study, using a very similar methodology, showed that the use of 7% MA followed by 2% CHX + 0.2% CTR has a long-term antimicrobial effect [6]. However, to the best of our knowledge, the residual antimicrobial activity of mixed solutions of 7% MA + 2% CHX and 7% MA + 2% CHX + 0.2% CTR in final irrigation protocols is unknown. In this regard, it would be interesting to establish not only their antimicrobial effectiveness but also a possible synergic effect. From a clinical point of view, it is likewise useful to investigate combinations that help reduce the number of irrigating solutions used in root canal preparation. The aim of this study was therefore to determine the residual antimicrobial activity of several final irrigation protocols with 7% MA alone and combined with CHX, CTR or both, in root canals infected with *E. faecalis*.

## Results

There were no statistically significant differences between groups in *E. faecalis* counts before instrumentation (*p* = 0.978). Just after preparation, no bacteria could be isolated in the experimental groups (100% eradication at short-term) except for the control group, 0.9% SS, which exhibited growth the first day in all samples. All negative controls showed no bacterial growth throughout the study. Table 1 gives the number of grown samples, along with the minimum, maximum and median of the day of bacterial regrowth. The *p* values of pair-by-pair comparison between groups are also shown as footnote in the Table.

**Table 1:** **Irrigation protocols.** ***E. faecalis*** **counts counts before and after instrumentation (mean ± standard deviation)**

**Final irrigating solutions**	**Short-term**		**Long-term**				**Paired comparison** **
	**CFUs × 10** ^**3**^ **of** ***E. faecalis***		**Grown samples at 60 days**				
	**Before instrumentation** *	**After instrumentation**	**Number and % of grown samples**		**Minimum**	**Maximum**	
**Group 1:** 2.5% NaOCl	88 ± 69.07	0	12	100	3	20	a
**Group 2:** 7% MA	106 ± 135.6	0	11	91.7	5	>60	b
**Group 3:** 7% MA + 0.2% CTR	119 ± 156.1	0	7	58.3	5	>60	b,c
**Group 4:** 7% MA + 2% CHX	119.4 ± 154.5	0	5	41.7	24	>60	c,d
**Group 5:** 7% MA + 0.2% CTR +2% CHX	93.1 ± 84.1	0	4	33.3	9	>60	c,d
**Control group:** 0.9% Saline solution^+^	88 ± 69.07	0.004 ± 0.007	-		-	-	-

Number of grownsamples at 60 days. Median of the day of regrowth. n = 12 per group.

NaOCl: sodium hypochlorite. MA: maleic acid; CTR: cetrimide; CHX: chlorhexidine.

^**+**^No long-term analysis was performed because all samples grew on first day.

*Before instrumentation there was the same *E. faecalis* level in all groups determined by an ANOVA test previously subjecting data to a Poisson normalization transformation: √(basal data + 3/8) (*p* = 0.978).

**The same letter shows differences that were not statistically significant determined by the Log-Rank test (statistical significance level was set at *p*< 0.05). *p* values of pair by pair comparisons: group 1 *vs* group 2: *p =* 0.015, group 1 *vs* group 3: *p* = 0.010, group 1 *vs* group 4: *p*< 0.001, group 1 *vs* group 5, *p*< 0.001, group 2 *vs* group 3: *p* = 0.247, group 2 *vs* group 4: *p* = 0.005, group 2 *vs* group 5: *p* = 0.005, group 3 *vs* group 4: *p* = 0.193, group 3 *vs* group 5: *p* = 0.157, group 4 *vs* group 5: *p* = 0.928.

**Figure 1:**
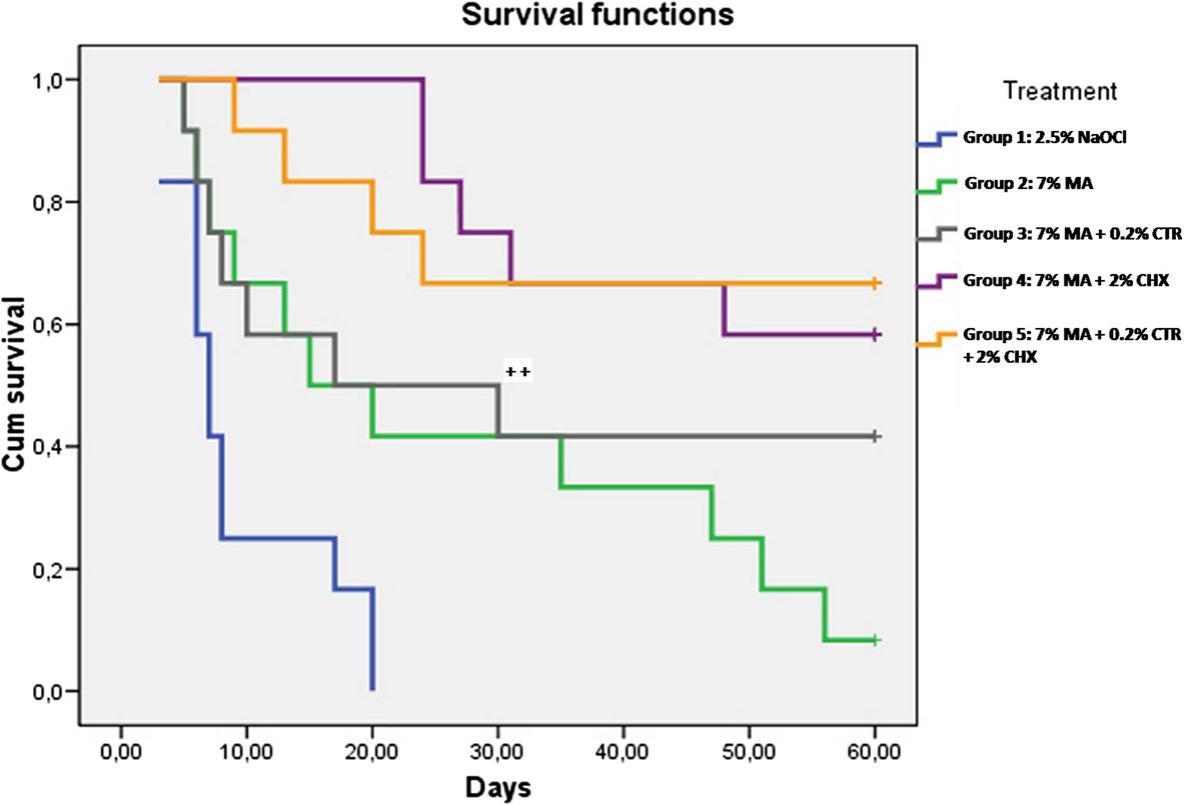
**Kaplan-Meier survival probabilities at 60 days (probability of no growth) for experimental groups.** Cum survival: percentage of samples that did not show *E. faecalis* growth at a given time. For clarity: ++, 7% MA + 0.2% CTR. At day 30, there was growth of *E. faecalis* in 7 out of 12 samples, meaning that 41.67% survived without growing.

At 60 days, all samples in the 2.5% NaOCl group showed regrowth, exhibiting statistically significant differences with respect to all the other study groups. The 7% MA group, with regrowth of 91.66% of samples, showed significant differences with respect to the groups in which MA was combined with CHX (41.66%) and CHX + CTR (33.33%), but not when combined with only CTR (58.33%). The association of MA with CHX and with CHX + CTR showed the best results, with a very low number of regrown samples at 60 days (respectively, 5 and 4 samples out of 12). The results of Kaplan-Meier survival analysis, (excluding 0.9% SS group), are indicated in Figure 1 and its footnote.

## Discussion

The success of endodontic treatment depends largely on the control of microorganisms present in infected root canals [19]. The purpose of this study was to evaluate the efficacy of several irrigating solutions in the treatment of necrotic and infected root canals with *E. faecalis.* From a clinical standpoint this situation requires the use of chelating agents and solutions with residual antimicrobial activity. Given its demonstrated efficacy against *E. faecalis* biofilm, we used maleic acid as chelating, alone or combined with solutions of proven antimicrobial substantivity. These are facultative anaerobic gram-positive bacteria often selected for experimental studies [20] because they are frequently isolated from root canals in cases of failed endodontic treatment [21,22]; they can penetrate deeply into dentinal tubules, making their complete elimination difficult. In addition, *E. faecalis* may grow as biofilm, even in situations where nutrients are scarce [23], which increases their resistance in root canal walls [24].

At baseline, all groups showed a similar *E. faecalis* infection level, which is important in order to standardize the groups. Just after instrumentation it was not possible to isolate bacteria in any specimen except for the control group, but the posterior regrowth indicates that eradication was not complete. The presence of bacterial concentrations lower than the detection limit of the culture method may lead to false-negative counts [25]. For this reason, long-term follow up is essential. The survival analysis takes into account the entire time period (60 days), not just one or more points in time.

The use of chelating agents to eliminate the inorganic smear layer produced during instrumentation is an important step in root canal preparation. This layer harbours bacteria and can be detrimental to effective disinfection in the root canal wall and in dentinal tubules [26]. Also, final irrigation protocols with a decalcifying agent seem advisable to increase the bond strengths of epoxy resin-based and methacrylate resin-based sealers to root canal walls [27-29]. Moreover, the addition of detergents to disinfecting solutions increase their antibacterial effects against *E. faecalis* in the dentinal tubules [30] and irrigation protocols with chelating agents combined with CTR [10] or CHX combined with CTR [6] are an alternative to the use of NaOCl as final irrigating solutions.

The results of the present study support the greater residual activity of the solutions that use MA as opposed to a solution of 2.5% NaOCl as the final irrigant root canal preparation. Overall, it was shown that the incorporation of antimicrobial agents to MA enhances its residual activity. Thus, MA + CTR at 60 days showed a lower number of regrown samples (7 out of 12) *versus* MA alone (11 out of 12), confirming previous results [10]. On the other hand, the best results were obtained with protocols combining 7% MA with CHX or CHX + CTR, which achieved very prolonged residual antimicrobial activity. At 60 days, in no case did the regrown samples reach 50% (median > 60). These results were partly expected in view of the substantivity [8] of the antimicrobial solutions. In fact, CHX has been documented to remain in the root canal dentin in antimicrobially effective amounts for at least 12 weeks [31]. Also, the availability of 2% CHX when mixed with MA is only slightly reduced [18], and the associated use of CTR and CHX has been established to provide better results than their applications as single agents against *E. faecalis* biofilms [32]. Notwithstanding, studies that involve polymicrobial biofilms and/or root canals with complex anatomic configuration are needed to confirm the efficacy of these combined solutions.

## Conclusion

In conclusion, under the conditions of this research study and given the key importance of effective residual antimicrobial activity in infected root canals, the use of 7% MA mixed with 2% CHX or 2% CHX + 0.2% CTR can be recommended for final irrigation protocols. Further studies are needed to evaluate the disinfection efficacy of these mixed solutions with contemporary irrigant agitation techniques [33] as compared to conventional syringe needle irrigation.

## Methods

The protocol followed in this study was approved by the Ethics Committee of the University of Granada, Spain. Eighty-four single-rooted anterior human teeth stored in 0.1% thymol solution at 4°C were decoronated to obtain roots 12 mm in length. To allow handling of the tooth during the instrumentation sequence of the experiment, a customized model of each tooth was fabricated with polyvinyl-siloxane impression material (Zhermack, Rovigo, Italy) [6]. Each root and its corresponding customized tooth model were autoclaved at 121°C. Then each root was placed in a 1.5 ml Eppendorf tube, immersed in sterile brain-heart infusion broth (BHI) (Scharlau Chemie S.A., Barcelona, Spain), and sealed and incubated for 1 week at 37°C. The specimens were inspected daily to ensure that the BHI broth showed no signs of turbidity. From this stage forward, all specimens were processed using strictly aseptic protocols.

### Contamination with Enterococcus faecalis

From a subculture of *E. faecalis* (ATCC 29212), a 1 McFarland standard suspension was prepared in BHI and then diluted 30-fold to obtain an initial bacterial suspension of approximately 1 × 10^7^ colony-forming units per millilitre (CFU ml^−1^). Afterwards, 1.2 ml of this suspension and the sterilized tooth were added to an Eppendorf tube and were incubated for 4 weeks under aerobic conditions at 37°C, with reinoculation performed every 7 days. The cultures were checked for purity by gram stain and colony morphology.

The working length was established for each tooth using a #15 K-file (DentsplyMaillefer, Ballaigues, Switzerland), and the apices of the teeth were sealed with cyanoacrylate. Each root was inserted into its customized model, and the interface between the outer tooth surface and impression material was sealed with cyanoacrylate.

Samples were taken from the root canal for bacterial counts before and after preparation with the sterile #15 K-file (DentsplyMaillefer, Ballaigues, Switzerland) placed in the canal to within 1 mm of working length, and the canal was circumferentially filed for 30 sec. Three consecutive sterilized paper points were introduced into the canal to absorb the BHI broth during 1 min. The paper points and the K-file were transferred to an Eppendorf tube containing 0.5 ml of BHI broth and vortexed for 30 sec for serial dilutions. For quantitative bacterial assessment, each dilution was seeded on plates containing BHI agar medium, and they were incubated at 37°C for 48 hrs, at which time the CFUs were counted.

### Root canal preparation

The root canals were instrumented using the ProTaper system (DentsplyMaillefer, Ballaigues, Switzerland) to the working length to up to a F3 master apical file (DentsplyMaillefer, Ballaigues, Switzerland) following the manufacturer’s instructions. During instrumentation, the experimental groups were irrigated with 6 ml of 2.5% NaOCl and the control groups with 0.9% saline solution (SS). A 2.5% concentration was selected because it is less toxic than 5.25% [34], but it has demonstrated antimicrobial activity against *E. faecalis* biofilm in dentin [9].

A total of 72 specimens were divided into 5 experimental groups according to the final irrigating solution (Table 1). Group 1: 2.5% NaOCl; Group2: 7% MA; Group 3: 7% MA + 0.2% CTR; Group 4: 7% MA + 2% CHX; Group 5: 7% MA + 0.2% CTR + 2% CHX; and Control group: 0.9% saline solution. The final irrigation volume was of 5 ml. Twelve roots (2 per group) that were instrumented and not infected served as negative or sterility controls. Irrigation was carried out using a 3 ml Luer-Loc syringe coupled to a 30-gauge needle tip placed passively into the canal up to 2 mm from the apical foramen without binding. In all study groups, the final irrigation solution remained in the root canal for 1 min and then the root canals were dried with sterile paper points. After instrumentation, the root canals were filled with BHI broth. Samples were then collected as described above (to obtain the initial sample), and the results were expressed as CFUs.

### Regrowth determination

When no bacteria were collected after the instrumentation, the specimens were refilled with the same broth and samples were collected daily for 60 days. All collected samples were incubated for 24 hrs at 37°C. Turbidity was recorded as an indicator of bacterial growth in the root canal. Once turbidity was present, a sample of the turbid broth was streaked onto blood agar plates, and bacteria were identified to ensure that there was no contamination other than *E. faecalis.* At this point, the specimens were considered regrowth or positive.

### Statistical analysis

To compare at baseline the *E. faecalis* counts between groups, an ANOVA test was performed, previously subjecting data to a Poisson normalization transformation using the formula: $$ \sqrt{\left(\mathrm{basal}\ \mathrm{data}+\raisebox{1ex}{$3$}\!\left/ \!\raisebox{-1ex}{$8$}\right.\right)} $$. Final sample growth status and the associated survival times in days (up to 60 days) were considered as follows: censored status when the sample had not grown (i.e., a survival time of 60 days); failed status when the sample had grown, the survival time being the number of days from the beginning. Cumulative survival proportions (samples without *E. faecalis* regrowth) were evaluated using non-parametric Kaplan-Meier survival analysis. Differences among groups were tested using the log-rank test and the level of statistical significance was set at *p*< 0.05. All statistical analyses were performed by means of SPSS 17.0 software (SPSS Inc., Chicago, IL).
